# Gaps and Interventions across the Diagnostic Care Cascade of TB Patients at the Level of Patient, Community and Health System: A Qualitative Review of the Literature

**DOI:** 10.3390/tropicalmed7070136

**Published:** 2022-07-15

**Authors:** Harsh D Shah, Mahalaqua Nazli Khatib, Zahiruddin Quazi Syed, Abhay M. Gaidhane, Sandul Yasobant, Kiran Narkhede, Priya Bhavsar, Jay Patel, Anish Sinha, Tapasvi Puwar, Somen Saha, Deepak Saxena

**Affiliations:** 1 Department of Public Health Science, Indian Institute of Public Health Gandhinagar (IIPHG), Gandhinagar 382042, India; yasobant@iiphg.org (S.Y.); knarkhede@worldbank.org (K.N.); priyabhavsar@iiphg.org (P.B.); jpatel@iiphg.org (J.P.); asinha@iiphg.org (A.S.); tpuwar@iiphg.org (T.P.); ssaha@iiphg.org (S.S.); ddeepak72@iiphg.org (D.S.); 2 Global Evidence Synthesis Initiative, School of Epidemiology and Public Health, Jawaharlal Nehru Medical College, Datta Meghe Institute of Medical Sciences, Wardha 442004, India; nazlikhatib@dmimsu.edu.in (M.N.K.); zahirquazi@dmimsu.edu.in (Z.Q.S.); abhaygaidhane@dmimsu.edu.in (A.M.G.)

**Keywords:** diagnostic gaps, care cascade, tuberculosis, review

## Abstract

Tuberculosis (TB) continues to be one of the important public health concerns globally, and India is among the seven countries with the largest burden of TB. There has been a consistent increase in the notifications of TB cases across the globe. However, the 2018 estimates envisage a gap of about 30% between the incident and notified cases of TB, indicating a significant number of patients who remain undiagnosed or ‘missed’. It is important to understand who is ‘missed’, find this population, and provide quality care. Given these complexities, we reviewed the diagnostic gaps in the care cascade for TB. We searched Medline via PubMed and CENTRAL databases via the Cochrane Library. The search strategy for PubMed was tailored to individual databases and was as: ((((((tuberculosis[Title/Abstract]) OR (TB[Title/Abstract])) OR (koch *[Title/Abstract])) OR (“tuberculosis”[MeSH Terms]))) AND (((diagnos *) AND (“diagnosis”[MeSH Terms])))). Furthermore, we screened the references list of the potentially relevant studies to seek additional studies. Studies retrieved from these electronic searches and relevant references included in the bibliography of those studies were reviewed. Original studies in English that assessed the causes of diagnostic gaps and interventions used to address them were included. Delays in diagnosis were found to be attributable to both the individuals’ and the health system’s capacity to diagnose and promptly commence treatment. This review provides insights into the diagnostic gaps in a cascade of care for TB and different interventions adopted in studies to close this gap. The major diagnostic gaps identified in this review are as follows: people may not have access to TB diagnostic tests, individuals are at a higher risk of missed diagnosis, services are available but people may not seek care with a diagnostic facility, and patients are not diagnosed despite reaching health facilities. Therefore, reaching the goal to End TB requires putting in place models and methods to provide prompt and quality assured diagnosis to populations at par.

## 1. Introduction

Tuberculosis (TB) is an ancient infectious disease that kills more people than other such diseases. During the COVID-19 pandemic, the most obvious impact was significantly reducing newly diagnosed TB cases and their reporting. According to World Health Organization (WHO) estimates, approximately 9.9 million people were infected with TB, and about one and a half million died from it in 2020 [1]. Over 95% of cases, as well as deaths, are from developing nations. Though TB is curable with affordable treatment, the incidence of TB has been falling steadily during these last ten years at a rate of merely 1–2% every year. The substantial reduction in TB case detection and reporting after the outbreak of COVID-19 in 2019 probably reflects both supply and demand-side disruptions to TB diagnostic and treatment services [2,3]. Delay in the diagnosis of TB continues to be the key challenge in successfully managing TB, particularly in developing nations with a high burden of disease.

TB continues to be India’s severest health emergency. India is among the seven countries with the largest burden of TB. In India, an estimated 2.59 million new cases of TB crop up every year, and an estimated half million Indians die yearly due to TB [2]. Studies have shown that timely and correct diagnosis and recognition of drug-resistant TB is essential in reducing the disease burden. Adequate and quality diagnostic care has resulted in better outcomes regarding incidence, prevalence, and mortality related to TB [3,4,5]. However, the underdiagnosis of TB has challenged its management and affected these outcomes adversely [6,7,8].

There has been a consistent increase in the notifications of TB across the globe. However, the estimates revealed a gap between the incident and notified TB cases, indicating a considerable number of patients who remain undiagnosed or ‘missed’ [1]. India, Nigeria and Indonesia account for 46% of all the ‘missed’ people with TB [9]. Most of this population remains either undiagnosed or poorly diagnosed [2]. It is crucial to understand who is ‘missed,’ find this ‘missing’ population and provide quality care. To reach the ‘missing’ population, addressing quality of care and planning user-centric strategies is necessary [10]. Often, there is a significant delay in diagnosis. Findings of a meta-analysis revealed a delay of more than three weeks in diagnosing TB in Ethiopia [11].

To improve TB outcomes, the overall functioning of the health system, particularly in countries with a high burden of TB, has to be improved, with priority toward better diagnosis [5,6]. Therefore, all patients with TB must have access to diagnosis and care. Improving access to quality services, active case-finding strategies, and other approaches have been suggested to fill the gap in the care cascade for TB. Despite these strategies, innovations in equipment and treatment, and scale-up of TB care services, gaps in the cascade of care persist, and control over the disease is far from achieved [10]. Measures for new diagnostics, medications, and vaccines are improving, but slowly. The WHO End TB Strategy launched in 2015 calls for fast-tracking decreases in burden with 50% reductions in the incidence of TB in 2025 and 90% reductions in the incidence of TB in 2035. It also calls for 75% reduction in mortality due to TB in 2025 and a 95% reduction in mortality due to TB in 2035 [12,13]. A modelling study indicated that the current strategies for controlling TB would not be adequate to achieve India’s End TB strategy 2025 milestones [14,15]. To achieve this milestone, the Lancet Commission on TB has recommended funds for established policies [15,16].

In view of these complexities, we reviewed the diagnostic gaps in the care cascade for TB and the interventions proposed by studies to fill these gaps. We have also tried to identify research gaps to guide theory-informed intervention development and help public health and policymakers design approaches to fill the diagnostic gaps in TB care. However, these priorities are not general and differ by country.

## 2. Methodology

### 2.1. Criteria for Considering Studies for This Review

We included studies providing data on diagnostic gaps in the TB care cascade and suggested interventions irrespective of study designs and publication status. Studies providing information on tuberculous patients regardless of age, gender, ethnicity, and type of TB were included. Original studies in English that assessed the causes of diagnostic gaps and interventions used to address them were included. Because of resource limitations, we did not include studies published in other languages and excluded book chapters, book reviews, commentaries, correspondence, and letters to the editors.

### 2.2. Search Methods for Identification of Studies

We searched Medline via PubMed and CENTRAL databases via the Cochrane Library. The search strategy was tailored to individual databases and was as follows: ((((((tuberculosis[Title/Abstract]) OR (TB[Title/Abstract])) OR (koch *[Title/Abstract])) OR (“tuberculosis”[MeSH Terms]))) AND (((diagnos *) AND (“diagnosis”[MeSH Terms])))). Furthermore, we screened the references list of the potentially relevant studies to seek additional studies. Studies retrieved from these electronic searches and relevant references included in the bibliography of those studies were reviewed. The study variables associated with diagnostic care cascade gaps, delay and missed diagnosis of TB from the patient or system side, gaps in delivering the services due to access or availability, and multidimensional factors related to the type of TB patients were identified during the review. Further, the search was done to identify the relevant reports and guidelines to support the search strategies.

### 2.3. Selection of Studies and Data Extraction

The review of the studies was scheduled in two phases. In the first phase, three reviewers searched the studies from the database. In the second phase, the retrieved studies were reviewed by two reviewers initially from the titles, abstracts, and available full texts. Another reviewer also reviewed approximately 20% of these studies to validate the inclusion of studies. Disagreements were resolved through discussion. The retrieved studies’ data on TB diagnostic gaps and interventions were assessed for inclusion using the Rayyan screening app [17]. Data were extracted from all the studies that met the inclusion criteria.

## 3. Findings

### 3.1. General Characteristics of Reviewed Studies (Search Results)

**Figure 1:**
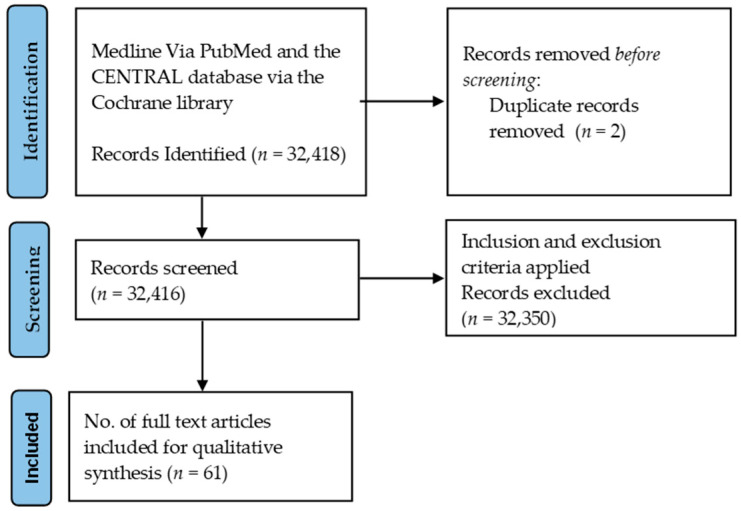
Identification of search strategies for qualitative review synthesis databases and registers on diagnostic gaps in TB patients.

We retrieved 32,408 records from the database and 10 records from different other sources. There was a total of 32,416 records with non-duplicated hits. After screening by title, abstract and full-text availability, eighty-seven articles remained. Sixty-six studies were finally included for the synthesis of evidence in this review based on inclusion and exclusion criteria (Figure 1). The review of studies was carried out from the final list of quantitative, qualitative, and mixed-method studies to ensure the qualitative evidence synthesis. Additional electronic searches, a bibliography of relevant studies and hand searches yielded 54 studies. Most of the research has been focused on clinical outcomes; only a few studies have scrutinized the impact of TB on patients’ quality of life.

Studies have reported that some people with active TB do not have access to TB diagnostic facilities. In contrast, others may have access to diagnostic facilities but still are not successfully diagnosed [18,19,20,21]. Diagnosis of TB and early initiation of treatment is vital to stop transmission [8,12,22]. However, despite the high burden of TB, active case finding (ACF) is frequently not executed, causing delays in diagnosis and treatment [8]. In India, snowballing case detection and diagnosis of new patients of TB using new diagnostic tests is vital to better outcomes in a cascade of care, particularly for some types of TB, such as smear-negative and multi-drug-resistant (MDR) TB [18]. The ‘Detect-Treat-Prevent-Build’ (DTPB), an Indian approach of the National Strategic Plan 2017–2025 to eliminate TB, aims to detect all drug-sensitive and drug-resistant TB cases, especially those seeking care from private practitioners and undiagnosed cases of TB in the high-risk population [2].

### 3.2. Diagnostic Gaps and Possible Solutions

Delay in diagnosis waw attributable to both the individuals and the capacity of the health system to diagnose and commence treatment promptly [23]. We have summarised the gaps in diagnosis in the ‘cascade of care’ for TB and interventions suggested by studies as per the following (Table 1):

1. People may not have access to TB diagnostic tests.
2. Individuals with a higher risk of TB missed the complete diagnosis algorithm.
3. Services are available, but people may not seek care from a diagnostic facility.
4. Patients may not get diagnosed with TB, despite reaching health facilities.

#### 3.2.1. Gap: People May Not Have Access to TB Diagnostic Tests

Individuals with TB may not have access to TB diagnostic tests, and the fact that people do not survive due to this limitation is most disturbing. Some areas still exist where approved diagnostic facilities are inaccessible or practicable to implement. Marginalised or internally displaced (including native people residing in the Amazon of Brazil and Peru, and in parts of Ethiopia, rural India, etc.) and high-burden sub-populations (slum dwellers, prisoners, etc.) have predominantly been deprived of access to TB care services [11,24,25,26,27,28,29]. This is a sizeable gap and may significantly cause the burden of TB. Recognising such marginalised sub-populations with deprived access to TB care services is the first step to decreasing this gap. Tailoring approaches targeted toward rural areas can improve the timely detection of TB [30]. Improving the accessibility of TB care services in geographical regions not connected to health services can be achieved through deploying health extension workers [31].

A Cochrane systematic review indicates that door-to-door screening of patients for active TB and conducting TB diagnostic camps near the residence and people’s workplaces may enhance the detection of cases in the locations with a high prevalence of undiagnosed TB [32]. Enhancing national TB programme visibility through community awareness about TB, predominantly in marginalised, deprived, and rural populations can support timely diagnosis and management [11,14]. India’s 2017 national strategic plan prioritises continued, systematic screening of high-risk subpopulace, such as prison inmates, miners, migrants, refugees, urban slum dwellers, and tribals with a high TB burden [2,33]. Active private sector involvement through a patient support system can fill this gap. The inclusion of spiritual leaders, traditional healers, prominent clans or religious leaders through TB programme awareness workshops has shown significant results in identifying presumptive cases and linking them to health facilities [29,34,35,36,37]. Active screening at camps for marginalised populations has also increased case notification [32,33]. Distance or other access barriers [38] and the unavailability of diagnostic care services for extrapulmonary TB [23,39] can also lead to delays in diagnosis. Improving access and guaranteeing the availability of approved diagnostic facilities, particularly for EP-TB, can help in reducing this gap [20].

In many low-middle income countries (LMICs), community health workers (CHWs) in the public sector represent the point of initiation of care [40]. CHWs can screen individuals with presumptive TB symptoms, collect sputum samples, and refer patients with suspected TB cases; however, they are not trained to diagnose TB [40]. As per the national policy, CHWs and dispensaries in many countries are required to refer patients from public-sector community-based facilities to higher primary and secondary health facilities. However, insufficient enticements and health systems allow steady referral [2]. A study in an Indian setting has reported that deploying CHWs trained to recognise TB symptoms improved ACF by mobilising the community and enabling the delivery of health services across several national programmes at the doorstep [41]. Another study from Mozambique has found that engaging CHWs to encourage local facility-based screening of all individuals arriving for care increased case notification [42]. Modelling studies [43,44] have reported that such types of interventions can reduce the incidence of TB.

In India, up to 40% of patients with symptoms suggestive of TB visit chemists or drugstores as the first point of health care [45]. However, they are rarely referred for screening and diagnostic services. A mixed-methods intervention study in an Indian setting implemented a multidimensional TB screening and referral intervention utilising private pharmacies involved in a public-private mix (PPM) programme. The study found that intervention significantly improved the diagnosis of TB, microbiological testing and test confirmation [45]. The study recommended functioning with current PPM programmes, particularly in societies where the pharmacy is the first contact, and using a combination of incentives custom-made to commercial and health mandates [45].

#### 3.2.2. Gap: Individuals with a Higher Risk of TB Missed the Complete Diagnosis Algorithm

Some groups, such as people with HIV (PLHIV) or immunosuppressed for other reasons, children, pregnant women, households and other close contacts of people with TB, and people who previously had TB are at higher risk of missed diagnoses [46,47]. People falling into high-risk groups are more prone to suffer from extrapulmonary TB, which is more challenging to diagnose. It requires biopsies and diagnostic tests (on non-sputum specimens) with low sensitivity [48].

The Lancet Commission recommends reaching populations at high risk for TB and bringing them into care with access to affordable, high-quality diagnostic services [15,16]. Current strategies for controlling TB focus on passive case-finding approaches and have proven inadequate in accomplishing goals for reducing incidence and mortality. ACF strategies can sidestep the challenges by bringing TB screening across the last mile to high-risk individuals. WHO strongly recommended that active screening should be concentrated on PLHIV, household contacts of TB patients, and workers exposed to silica dust [49]. However, the report does not reinforce the implementation of ACF in the general populace. Active screening can comprise a blend of identification based on symptoms, chest X-rays (CXR), sputum microscopy, or rapid molecular testing conducted outside healthcare facilities using mobile vans, camps, etc. Active screening of household contacts and contact tracing of TB patients is contemplated as a local form of spatial targeting [50,51].

Additionally, screening strategies considering individual-level risk factors and active screening within geographically-limited populations such as neighbourhoods or sub-districts can also prove effective [52]. Hence, focused ACF strategies, especially engaging facility-based CHWs, can increase case detections and enhance the momentum towards meeting the WHO’s End TB Strategy [15]. Utilising CHWs to encourage facility-based screening of household contacts of TB has improved TB notification in Mozambique [42]. This high-risk populace needs access to inexpensive, high-quality diagnostic services wherever they seek care. Investments are required to strengthen the capacity of the health system to deliver upfront advanced diagnostic testing facilities, which can minimise the duration of awaiting a complete diagnosis.

#### 3.2.3. Services Are Available, but People May Not Seek Care from a Diagnostic Facility

Some of the reasons for not seeking care and discrepancies in care-seeking behaviour are lack of awareness, lack of care-seeking behaviour, and challenges in navigating between health facilities [11,53]. Factors leading to delayed healthcare-seeking behaviour are inherent to the patient, family, community or related to the healthcare system but differ through different political, geographical, and socio-cultural settings [29].

Meta-analysis of Ethiopian studies showed that the delay was more significant in patients with poor knowledge about TB [11]. Often, people are not aware of free health services or are discouraged by lengthy waiting periods and undesirable visiting hours [54,55]. Many people also believe that government health services are not of good quality [54,55]. Addressing people’s knowledge gaps through community awareness programmes [23,56] and adopting intensive, state-of-the-art, and multidimensional interventions to support active case finding can dramatically improve local TB epidemics [3,29]. In one such programme, an awareness campaign and cell phone-based provisional cash transfers to all people screened at private clinics doubled TB notifications [55]. The success of this combined strategy depicts a model for related pooled interventions in other regions. However, judgments to scale-up of such pooled interventions entail estimations of their expected effect on the TB epidemic in that populace. A study from Zimbabwe adopted a mobile van-based active case-finding strategy, reporting a 40% reduction in the prevalence of TB [57].

Another reason for not seeking care with health facilities is that patients may not have care-seeking behaviour [56]. Public education strategies via TV, radio, or other electronic social media can modify this behaviour. Furthermore, putting health policies and practices in place can shield patients against the stigma and discrimination associated with the disease [15]. Being in contact with the TB patient can also lead to TB infection or having a TB itself. These contacts and asymptomatic patients may be recognised using CXR or biomarker-based screening as part of ACF.

#### 3.2.4. Gap: Patients Do Not Get Diagnosed Regardless of Reaching Health Facilities

Government reports and some studies from Indian [18] and South African [58] settings suggest that a substantial fraction of TB patients reach the health care facilities and access TB diagnostic tests but are not successfully diagnosed with TB [18]. Assessing this gap is particularly helpful for smear-negative and DR-TB, which are hard to diagnose. This gap points to the diagnostic gaps due to the usage of suboptimal diagnostic tests (e.g., sputum microscopy), lack of speciality services, knowledge and behaviour of HCPs, attitude and behaviour of patients, and poor adherence to programmatic diagnosis algorithms. Low TB testing rates by health care professionals (HCPs) can also cause diagnostic delays. Promoting TB testing rates may necessitate backing up HCPs, including auxiliary health personnel such as pharmacists, through public-private collaborations or incentives [45,59].

The additional challenge in the TB cascade of care is that for active TB, some frequently used diagnostic tools have relatively low (sputum microscopy) or higher but imperfect (Xpert MTB/RIF) sensitivity [60,61]. Some countries still rely on conventional low-cost tests with comparatively poor sensitivity (40% to 60%), like sputum microscopy as the primary diagnostic tool [61]. That may also lead to an incomplete diagnosis for the patient who may have drug-resistant TB. It is low-cost and can be undertaken in labs attached to primary healthcare facilities. More sensitive tests for TB, such as culture and tests for drug resistance, have been carried out at higher centres or reference labs located in areas not accessible to some populations. Xpert MTB/RIF has higher sensitivity (85% to 92%) but is sophisticated and expensive.

This test was originally recognised for diagnosing lung diseases in the populace with a high burden of HIV or MDR-TB. Subsequently, a recommendation came to replace microscopy with this assay as the first diagnostic test for TB, also helping diagnose various forms of EP-TB [2,3,4,5]. Some countries have made Xpert MTB/RIF available at the peripheral healthcare level, with care provided by CHWs [62]. However, patient pathways analyses depict that patients are probably not accessing these tests [40]. Apart from this, a wide-ranging inconsistency in Xpert MTB/RIF implementation can be addressed using appropriate diagnostic algorithms within the programme [63]. Regardless of incorporating Xpert MTB/RIF as the first-step diagnostic test for TB, the difference between incidence and notifications of TB is more than four million people. This points to the need for more accurate, rapid and cost-effective tools to increase the detection of TB cases [47]. A newer form of this assay with higher sensitivity and specificity, ‘Xpert MTB/RIF Ultra,’ has been launched [64].

Different forms of TB, such as pulmonary, extrapulmonary, drug-resistant TB and the possibility of recurrence of the disease, cause challenges in the TB care cascade. Using new TB diagnostic tests with higher sensitivity, such as same-day light-emitting diode microscopy [65] or automated nucleic acid molecular diagnostics [66] for rapid diagnosis and recognition of DR-TB through rapid susceptibility tests can reduce the gap [67,68,69]. Other diagnostic interventions under study comprise non-sputum-based detection with breath-based tests and computerised automated digital radiography [47]. Chest X-rays (CXR) can be triaged as screening tools for presumptive TB patients and possibly close the diagnostic gap using proper algorithms. However, CXR services are not extensively available in resource-limited settings. Advanced imaging tools, such as computed tomography and fluorodeoxyglucose PET-CT, remain beyond reach for many patients with TB. Authentication and valuation of newer artificial intelligence tools are ongoing, with many tools in the pipeline [63]. The lower sensitivity of new tools may be acceptable if found suitable for use in areas with poor access to laboratory health services. Evidence in hand suggests that the quality of care in the private sector falls short of international standards in a number of places and needs urgent attention [12]. Poor quality of diagnosis requires the upgradation of the entire healthcare system through utilising rapid, appropriate, accurate diagnostics and algorithms [28].

Many patients with DR-TB are lost due to misdiagnosis, low provider index of doubt, or challenges related to health facilities [56]. In most LMICs, private providers are the main point of healthcare [40]. The less-poor people seek care from formal and qualified providers, while the poor usually seek informal and unqualified providers. Most of the ‘missed’ people are understood to seek healthcare from private HCPs, including those who do not come under the umbrella of national TB programmes (NTPs), and were not an active part of policies that required screening, referral, or sample collection [12].

Qualitative studies have shown that HCPs often delay bacteriological testing for TB over pragmatic management, use inaccurate diagnostic tests, or do not test at all, leading to diagnostic delays. Attitude, behaviour, knowledge and skills of the HCPs also play a role in the diagnostic delay [20,70,71]. At government and private health facilities, patients are often not screened for symptoms or subjected to a diagnostic test; hence, many TB cases are missed [55]. Increasing TB testing rates, improving the capacity and skill-building of HCPs, and providing support through public-private initiatives have been used to tackle this issue. One significant factor is the varied, unsynchronised private health sector, the first point of contact for more than half of people with TB [72]. Evidence about the inappropriate diagnosis of TB cases among patients attending private clinics has raised concerns [40,73]. Various initiatives have been undertaken to efficiently involve the private sector in controlling TB, with differing success rates [73,74,75,76]. The EQUIP project in India demonstrated that the involvement of the private sector is feasible, with the capacity to provide noteworthy results to patients and the private/public sector by fostering proper performance of diagnosis and treatment [73]. The study recommended providing free or inexpensive access to cartridge-based nucleic acid amplification tests (CB-NAAT), such as GeneXpert, as the early diagnostic in private facilities for symptomatic TB patients [73].

## 4. Discussion

This review provides insights into the diagnostic gaps in a cascade of care for TB and different interventions adopted to close this gap. Delay in diagnosis continues to be a major challenge in TB control and prevention programmes in LMICs [35]. Reviewing diagnostic gaps in the TB care cascade that underlie poor outcomes and interventions required to close the gaps is vital to spur intervention development and framing of newer policies. These gaps can occur in the patient, family, society or healthcare system [38]. Hence, collaborative, person-centred and family-centred high-quality care can be used to fill the gap. The Lancet Commission recommends ending TB through reinforcing and intensifying our healthcare systems enabling successful implementation of established interventions [15,16]. Closing the know-do gap in numerous countries with a high burden of TB has been a persistent challenge, aggravated by a gap in technical facilities [77]. Effective and patient-centred diagnostic networks will help the countries meet the End TB targets [78]. Closing gaps in a TB cascade of care necessitates strategies directed at the level of patients, family, community, health system, and patient-HCP interface. Addressing gaps at various levels will necessitate a variety of intensive strategies such as community awareness and behaviour change campaigns, improved access to healthcare facilities, private sector interventions, high-quality rapid diagnostics, patient benefits schemes, capacity and skill building of HCPs, and scale-up of innovations. [2,15,16,38]. Increasing the budget allocation in diagnostic research, implementation research, and vaccines can produce meaningful returns [15].

Creating a TB-free world needs careful consideration of the failures of the past. The TB programme of South Africa achieved better outcomes in terms of population with access to a TB test but reported lesser treatment outcomes than India’s public sector TB programme [69]. The Government of India has earmarked funding to eradicate TB through the rapid decrease in disease burden and mortality by 2025 [2]. To accomplish this, the National Tuberculosis Elimination Programme (NTEP) has achieved noteworthy development in expansion of diagnostic and treatment services [79,80]; however, significant challenges in the healthcare system still exist. The private sector dominates the Indian healthcare system with the highest tuberculosis burden, where most patients seek care [21,71,81,82].

Countries need to address the diagnostic gaps through a patient-centric care approach to protect future generations from this preventable and treatable disease. The concerned government officials, national tuberculosis programmes, private clinics, non-governmental organizations and research-oriented pharmaceutical companies need to adopt essential remedial measures to do away with hindrances to eradicating TB [15]. Operational research on various areas, such as epidemiological and environmental factors and the capacity of the health system, should be encouraged to identify possible solutions and support the programme managers in customising people-centric policies.

### Study Limitations

Most studies included in this review were cross-sectional and undertaken in developing countries with the greatest burden of TB. Most studies included participants from different socio-economic strata and did not provide socio-demographic details. Explicit descriptions of these details of the participants in the included studies would have made it possible to extract similarities and differences between studies. This would have provided better insights for generalizability and transferability to other settings. The protocol was not registered anywhere.

## 5. Conclusions

A significant number of patients remain undiagnosed or missed, or there is a significant delay in the diagnosis of TB. This is attributable to both the individuals and the health system’s capacity to diagnose the cases promptly. The speed of progress is not sufficient to reach the SDG and the End TB Strategy goals. Reaching the goal of End TB requires putting in place models and methods to provide prompt, and quality assured diagnosis to populations at par. Activities required to speed up development towards global targets for reducing the burden of TB disease include closing the incidence–notification gaps, increasing the proportion of notified cases from public and private sectors that are bacteriologically confirmed and monitoring to ensure that people are correctly diagnosed and started on the most effective treatment regimen as early as possible. The TB programme has to review the existing laboratory networks, network optimisation, technology landscaping, the capacity of the health system to complete diagnosis with NAAT (nucleic acid amplification test for MDR/RR TB) testing with an appropriate system for collection and transport of samples and the implementation of follow up guidelines for those who are under treatment.

## Figures and Tables

**Table 1:** Identified diagnostic gaps and possible intervention matrix through qualitative review.

Sr. No.	Diagnostic Gaps	Reasons for Identified Gaps	Suggested Interventions
1	People may not have access to TB diagnostic tests	Marginalised populations or internally displaced or geographical distance and subpopulation Unavailability of diagnostic facilities in rural areas (especially for EP-TB) Insufficient referral mechanism at community-based facilities (Insufficient system and enablers in place)	Increasing and ensuring the availability of TB services in areas that are unconnected to health facilities using health extension workers Engaging the private sector, including informal providers Active screening at camps Improving access to the health facility for tests Ensuring referral mechanism at community-based facilities
2	Services are available, but people may not seek care with a diagnostic facility	Lack of awareness regarding TB Patients may not have a care-seeking behaviour Patients may be asymptomatic or have faced challenges in navigating between health facilities	Community awareness programmes for patients Multifaceted and innovative interventions to improve ACF Pubic education strategies for improving care-seeking behaviour Identify asymptomatic individuals (using CXR or biomarker-based screening)
3.	Patients do not get a complete diagnosis of TB, despite reaching health facilities	Low TB testing rates Use of suboptimal diagnostic tests Poor quality of diagnosis with limited capacity of laboratories Different policies at the private health facilities Poor adherence to diagnostic algorithms for the diagnosis Wide variability in the implementation of Xpert MTB/RIF Lack of specialist services in health facilities for EP-TB Attitude and behaviour of the HCPs HCPs often delay or defer bacteriological TB testing Limited knowledge and skills of HCPs (often use inaccurate diagnostic tests or omit tests) Incompetency of the doctor (suspecting and diagnosing)	Proper review system for increasing TB testing rates Public-private collaborations or provision of incentives to support HCPs Using more sensitive new TB diagnostic tests (LED microscopy or automated nucleic acid molecular diagnostics) Upfront Xpert MTB/RIF assay Facilitating the identification of DR-TB via rapid susceptibility testing Improving the public healthcare system (use of rapid, accurate diagnostics and algorithms) Use of appropriate diagnostic algorithms uniformly Capacity and skill-building of HCPs with responsive behaviour to the patients
4.	Individuals with a higher risk of missed diagnosis	PLHIV (immunosuppressed for other reasons) children People previously infected with TB Contacts of TB patients	Systematic screening of high-risk populations and contacts Longitudinal follow-up during treatment and of old TB patients

## Data Availability

All relevant data supporting this study’s findings are within the manuscript.
